# Phosphoproteomics-guided tau biomarker discovery in amyotrophic lateral sclerosis (ALS) and Alzheimer's disease (AD)

**DOI:** 10.3389/fnins.2025.1714196

**Published:** 2025-11-14

**Authors:** Nitesh Sanghai, Amir Barzegar Behrooz, Hamid Latifi-Navid, Javad Fahanik Babaei, Geoffrey K. Tranmer

**Affiliations:** 1 College of Pharmacy, Rady Faculty of Health Sciences, University of Manitoba, Winnipeg, MB, Canada; 2 Electrophysiology Research Center, Neuroscience Institute, Tehran University of Medical Sciences, Tehran, Iran

**Keywords:** Phosphoproteomics, neurofilaments, amyotrophic lateral sclerosis, Alzheimer's disease, neurodegenerative disease

## Introduction

Protein phosphorylation is a pivotal post-translational modification that regulates signaling networks and cellular functions (Ardito et al., 2017). Disruptions in kinase and phosphatase activity drive aberrant phosphorylation patterns, which are increasingly recognized as central to neurodegenerative diseases (NDDs) (Espargaró and Sabate, 2024; Tenreiro et al., 2014). Among these, tau hyperphosphorylation has long been established as a hallmark of Alzheimer's disease (AD), where it destabilizes microtubules and promotes the formation of neurofibrillary tangles (Noble et al., 2013). Remarkably, recent evidence now implicates phosphorylated tau in the pathology of amyotrophic lateral sclerosis (ALS), although distinct tissue-specific mechanisms (Abu-Rumeileh et al., 2025). This convergence suggests that tau phosphorylation is not only an AD signature but may represent a broader pathological axis across multiple NDDs (Mohallem et al., 2024). The advent of phosphoproteomics has transformed our ability to map disease-relevant phosphorylation sites with unprecedented resolution. By illuminating aberrant signaling cascades, phosphoproteomics provides powerful opportunities for biomarker discovery and therapeutic targeting (Morris et al., 2014). Herein, in this opinion, we discuss how phosphoproteomics-guided analysis of tau phosphorylation is redefining biomarker strategies in both AD and ALS, bridging two diseases once considered molecularly distinct.

Phosphorylated tau species, particularly p-tau217 and p-tau181, have emerged as robust diagnostic biomarkers in AD (Benussi et al., 2025). Benussi et al. (2025) demonstrated in a large clinical cohort that plasma p-tau217 levels effectively distinguished AD from frontotemporal lobar degeneration (FTLD). The p-tau 217/neurofilament light chain (NfL) ratio further refined the diagnosis. Their three-tiered plasma biomarker framework significantly diminished dependence on cerebrospinal fluid (CSF) analysis by more than 50%, highlighting the practicality of blood-based diagnostics for regular clinical application (Benussi et al., 2025).

While AD has long been defined by tau pathology, ALS has traditionally been viewed as a motor neuron disease. Recent findings, however, challenge this distinction. Abu-Rumeileh et al. (2025) documented increased serum p-tau217 and p-tau181 concentrations in ALS patients, which resemble markers of AD. Importantly, these phosphorylated tau variants were identified in muscle biopsies from ALS patients and localized to atrophic fibers using immunohistochemistry and mass spectrometry techniques. This peripheral source of p-tau challenges the CNS-centric perspective of tau pathology and raises intriguing questions about common phosphorylation mechanisms in both the central and peripheral nervous systems (Abu-Rumeileh et al., 2025).

**Figure 1:**
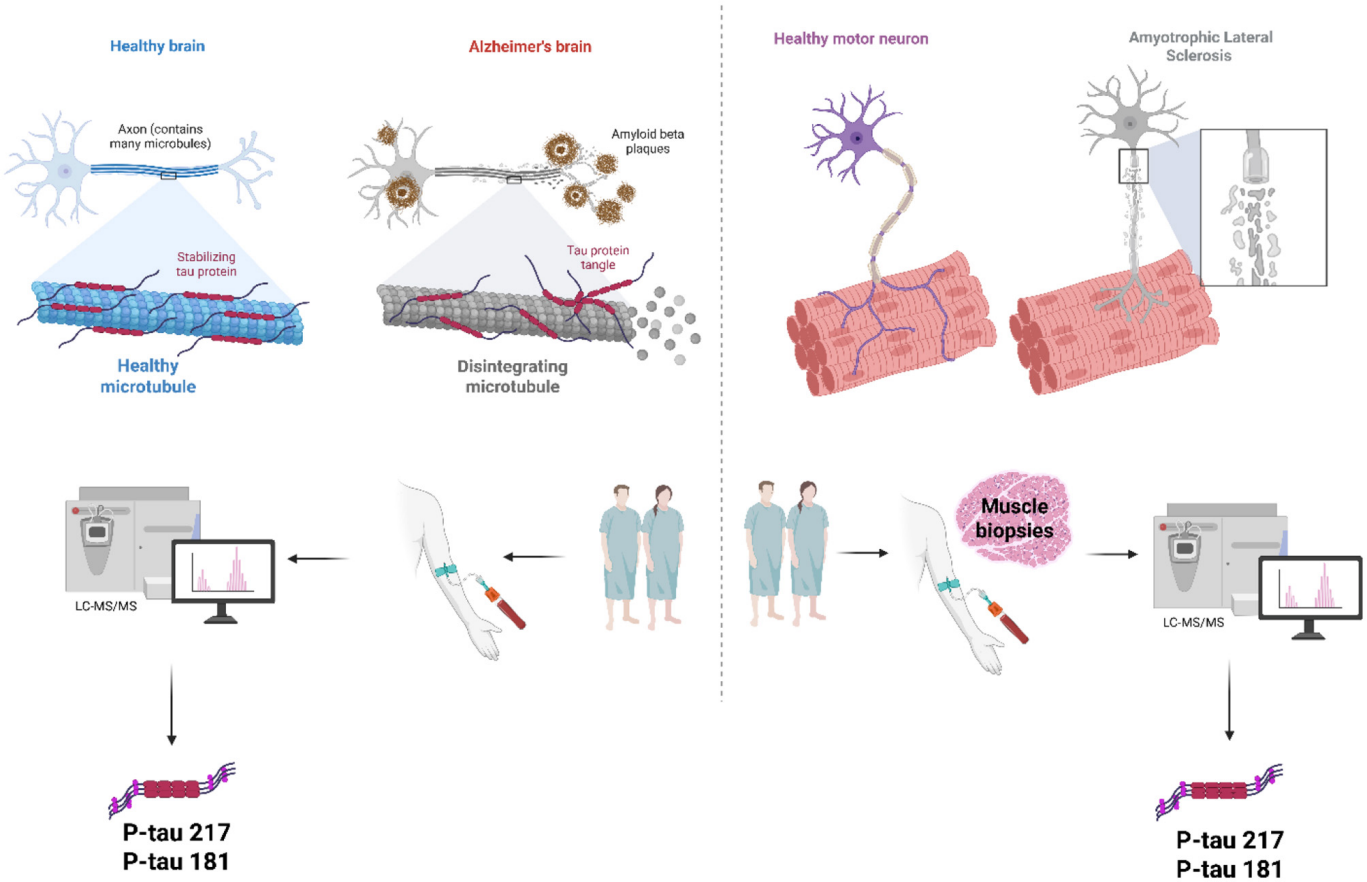
Phosphorylated tau as a molecular bridge between cognitive and motor neurodegeneration. Under normal circumstances, tau stabilizes microtubules; however, in Alzheimer's disease (AD), it becomes hyperphosphorylated, leading to the disintegration of microtubules and the formation of neurofibrillary tangles. In amyotrophic lateral sclerosis (ALS), tau pathology extends beyond the central nervous system (CNS), as phosphorylated tau species are detected in serum and muscle biopsies. These converging findings identify p-tau217 and p-tau181 as cross-disease biomarkers, connecting AD and ALS, and underscoring their translational potential for non-invasive diagnostics and precision medicine.

Phosphorylated tau has recently been identified as a unifying biomarker axis in AD and ALS, two conditions previously regarded as molecularly distinct. In AD, plasma p-tau217 and p-tau181 exhibit superior diagnostic accuracy relative to other biomarkers, with p-tau217 increasing early in the disease and demonstrating a robust correlation with amyloid deposition (Abu-Rumeileh et al., 2025; Kubota et al., 2025). Furthermore, incorporating plasma neurofilament light chain (NfL) ratios, a marker of neuroaxonal damage, enhances the test's specificity, as demonstrated in large clinical groups, thereby reducing the need for invasive CSF testing (Yuan and Nixon, 2021). Interestingly, concurrent advancements in ALS suggest that increased serum levels of p-tau217 and p-tau181 are associated with enhanced motor neuron dysfunction, increased clinical severity, and lower motor neuron (LMN)-dominant phenotypes. Even more surprisingly, phosphorylated tau species have been found in muscle biopsies from ALS patients (Abu-Rumeileh et al., 2025). This suggests that tau pathology can originate from outside the CNS. These findings collectively contest the conventional distinctions between AD and ALS, indicating a common framework for phosphoproteomics. P-tau217 is an emerging blood-based biomarker; specifically, it stands out as a promising cross-disease biomarker, serving as an early indicator of amyloid-related pathology in AD. However, it reflects LMN injury and kinase dysregulation in ALS (Abu-Rumeileh et al., 2025). Combining tau phosphorylation with markers like NfL could lead to multiplexed biomarker panels that can distinguish subtypes, differentiate between overlapping phenotypes, and inform cross-disease treatment plans (Figure 1).

**Figure 2:**
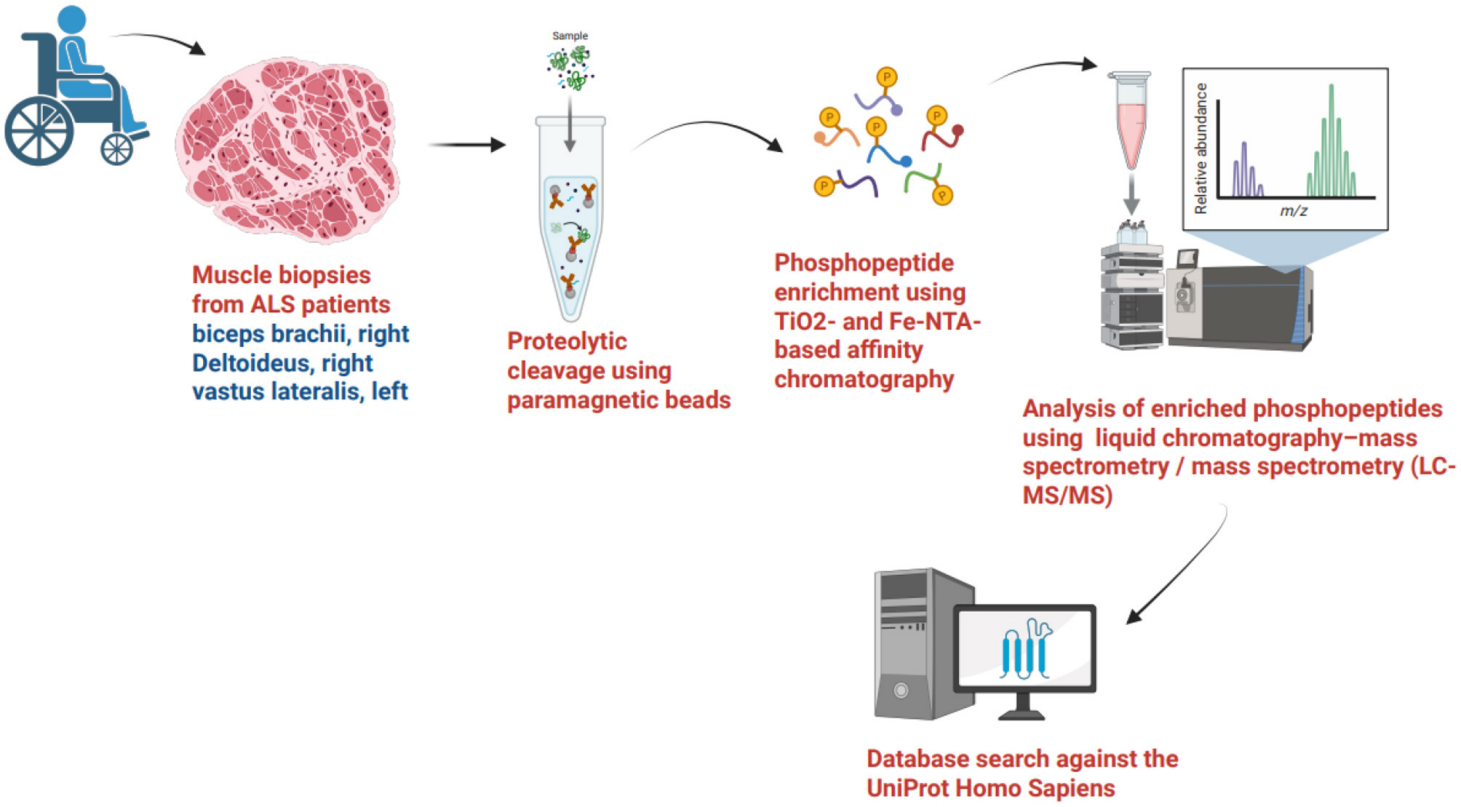
A brief overview of the phosphoproteomics workflow, including sample collection, protein extraction, phosphopeptide enrichment, and quantification, used in analyzing muscle biopsies with mass spectrometry methods.

Below is a brief overview of the phosphoproteomics workflow- including sample collection, protein extraction, phosphopeptide enrichment, and quantification utilized in the phosphopeptide analysis of muscle biopsies using Mass spectrometry (LC-MS/MS) methods (Figure 2).

Several emerging reports show that phosphoproteomics has translational potential (Muneer et al., 2025; Bekker-Jensen et al., 2020). Significant advances are being made in the clinical application of non-invasive plasma tests for p-tau181 and p-tau217 (Ashton et al., 2024; Zhang et al., 2024; Giacomucci et al., 2023). These developments could transform early diagnosis, aid in classifying subtypes, and inform the development of personalized treatments. But there are still significant obstacles to overcome. Since muscle-derived tau in ALS highlights the complexity of peripheral versus central biomarker sources of tau, it is essential to understand the peripheral tissue origin and specificity of phosphorylated tau in peripheral fluids, as muscle-derived tau may explain findings in ALS where central tau pathology is absent or less prominent. To facilitate reproducibility and clinical adoption of phosphoproteomics-guided biomarker detection, standardization of assays across platforms, sample processing, and reference ranges should be implemented concurrently. Large-scale, untargeted, discovery-based phosphoproteomics research, conducted longitudinally over time across different clinical stages of disease progression and diverse populations, is essential for future progress. Redefining tau phosphorylation as a continuum of pathology in neurodegeneration rather than as a disease-specific marker could be achieved by extending this framework beyond AD and ALS to other tauopathies, such as Parkinson's disease (PD) and mixed dementias.

## Strength of evidence by disease in both AD and ALS

Earlier studies have indicated that p-tau181 levels increase due to LMN involvement and degeneration, leading to higher p-tau181 in the plasma of ALS patients. However, some reports suggest that p-tau181 and p-tau217 may be released from peripheral axons and nearby denervated muscle fibers (Cousins et al., 2022). Additionally, pathological tau species have been detected in the blood and other peripheral tissues, such as skin and nerves, in patients with progressive supranuclear palsy, a tauopathy, which supports the presence of tau outside the CNS (Tanaka et al., 2024). This cumulative evidence suggests the peripheral involvement of tau release from the denervated muscle fiber and thus the peripheral involvement in the neurological conditions. However, further studies are warranted with a large number of cohorts, including patients from each stage of clinical progression in ALS because of the heterogeneous nature of the disease.

Both plasma p-tau181 (Chen et al., 2021) and p-tau217 (Mattsson-Carlgren et al., 2020) levels are reported to be increased in the early stages of AD or preclinical stages of AD. Both begin to rise in individuals who are cognitively normal but have early AD (defined by amyloid-beta positivity). Levels of p-tau181 and p-tau217 are higher in people with mild cognitive impairment and continue to increase over time, correlating with a decline in cognition and brain atrophy. The significant milestone is the recent approval of the development Elecsys pTau181 biomarker test, developed by two pharmaceutical companies, Roche in Basel, Switzerland, and Eli Lilly in Indianapolis, for the detection of AD (Rubin, 2025).

In case of ALS neurofilament light (NfL) and phosphorylated neurofilament heavy (pNfH) is elevated in presymptomatically and continue to rise in the early stages of the disease and recently regarded as promising prognostic and diagnostic biomarkers (Zucchi et al., 2020), nevertheless, no validated studies have shown the consistency of increase in p-tau181 and p-tau217 during the clinical progression of ALS starting from presymptomatic phase to symptomatic phase. The landmark study by Rumeileh and his team has caused a paradigm shift in how we think about peripheral involvement of taupathy in ALS. Additionally, patient stratification studies that combine neurofilament involvement stages with peripheral tau pathology might help solve the challenge of distinguishing between AD and ALS.

## Conclusion

Phosphoproteomics is transforming NDDs research by identifying both p-tau181 and p-tau217 as molecular links between AD and ALS. This connection opens the door for cross-disease biomarker strategies and the development of personalized therapies. Tau phospho-biology, therefore, serves as a unifying focus to address the complexity of currently untreatable NDDs. However, recent studies have prompted a paradigm shift in how we think about the involvement of peripheral tissues in NDDs. To tackle the challenges of overlapping diagnoses among different NDDs, we should consider the varied involvement of CNS and peripheral tissues at each stage of clinical progression, assessed longitudinally. Additionally, future large cohort studies with ALS and AD muscle biopsies are necessary to verify the findings of increased p-tau181 and p-tau217 using the reliable, reproducible, robust, and sensitive liquid chromatography-tandem mass spectrometry (LC-MS/MS) combined with data-independent acquisition (DIA) phosphoproteomics techniques.
